# Sustained conditional knockdown reveals intracellular bone sialoprotein as essential for breast cancer skeletal metastasis

**DOI:** 10.18632/oncotarget.2132

**Published:** 2014-06-26

**Authors:** Marineta Kovacheva, Michael Zepp, Stefan M. Berger, Martin R. Berger

**Affiliations:** ^1^ German Cancer Research Center (DKFZ), Toxicology and Chemotherapy Unit, Heidelberg, Germany; ^2^ Central Institute of Mental Health, Department of Molecular Biology, Mannheim, Germany

**Keywords:** breast cancer, skeletal metastasis, bone sialoprotein, conditional knockdown, BSP related signaling cascade

## Abstract

Increased bone sialoprotein (BSP) serum levels are related to breast cancer skeletal metastasis, but their relevance is unknown. We elucidated novel intracellular BSP functions by a conditional knockdown of BSP. Conditional MDA-MB-231 subclones were equipped with a novel gene expression cassette containing a tet-regulated miRNA providing knockdown of BSP production. These clones were used to assess the effect of BSP on morphology, proliferation, migration, colony formation and gene expression *in vitro*, and on soft tissue and osteolytic lesions in a xenograft model by three imaging methods. BSP knockdown caused significant anti-proliferative, anti-migratory and anti-clonogenic effects *in vitro* (p<0.001). *In vivo*, significant decreases of soft tissue and osteolytic lesions (p<0.03) were recorded after 3 weeks of miRNA treatment, leading to complete remission within 6 weeks. Microarray data revealed that 0.3% of genes were modulated in response to BSP knockdown. Upregulated genes included the endoplasmic reticulum stress genes *ATF3* and *DDIT3*, the tumor suppressor gene *EGR1, ID2* (related to breast epithelial differentiation), *c-FOS* and *SERPINB2*, whereas the metastasis associated genes *CD44* and *IL11* were downregulated. Also, activation of apoptotic pathways was demonstrated. These results implicate that intracellular BSP is essential for breast cancer skeletal metastasis and a target for treating these lesions.

## INTRODUCTION

Bone metastasis is an unfavorable event occurring in up to 70% of patients with advanced breast cancer. Severe bone pain, pathologic fractures, spinal cord compression, alteration of hematopoiesis by bone marrow infiltration and hypercalcemia are skeletal-related events caused by this malignant complication [1, 2]. Altogether, they have a decisive impact on the quality of life, morbidity and mortality of respective cancer patients [3]. Until today, metastasis renders breast cancer incurable, but deciphering the molecular mechanism of this process will help to improve the prognosis of affected patients.

Bone sialoprotein (BSP) has been associated with prognosis of breast cancer patients, since elevated serum levels were marker of subsequent bone metastasis and highly related to poor survival [4, 5]. BSP is a sialic acid-rich, phosphorylated glycoprotein, which is secreted and part of the non-collagenous extracellular organic matrix in human bone [4, 6]. It has an apparent molecular mass of 70-80 kDa including three polyglutamic acid domains, which confer hydroxyapatite-binding abilities [2, 5] and belongs to the small integrin-binding ligand N-linked glycoprotein family of proteins, which are clustered on human chromosome 4. Members of this family undergo a high degree of posttranslational modifications, which vary for a given protein in time (cellular differentiation) and space (tissue), and directly affect their biological functions [7]. Physiologically, BSP is synthesized by osteoblasts, osteoclasts, osteocytes and chondrocytes [2] and contains an Arg-Gly-Asp (RGD) sequence, which is a common recognition site for integrins [8] such as alpha-v-beta3 and alpha-v-beta5. The interaction of integrin alpha-v-beta3 with the RGD motif of BSP is related to cell attachment and plays a fundamental role in allowing BSP to engage with endothelial cells, osteoclasts and tumor cells [2]. BSP was found in primary malignancies such as breast [5, 9], prostate [10] and thyroid cancers [11]. Specifically, the BSP-mediated interaction between tumor cells and bone tissue was suspected to participate in the pathogenesis of bone metastasis [2, 5, 12, 13]. It is assumed that the secretion of BSP from tumor cells provides a selective advantage for their survival via binding to alpha-v-beta3 integrin and factor H, which protects them from complement – mediated lysis [13].

Based on these considerations, we hypothesized that BSP could serve as valuable target for anti-metastatic therapy [14, 15]. To study the effects resulting from the inhibition of BSP synthesis and secretion, we generated MDA – MB – 231 breast cancer cell clones containing a tet-regulated expression cassette with a specific miRNA against BSP and used Flp-recombinase-mediated cassette exchange (RMCE) for introducing the gene cassette into a well-defined genomic locus. Through this combination of the tetracycline-dependent gene regulation system with RNA interference (RNAi), temporal control of BSP production is established, avoiding compensatory changes on gene expression provoked by long lasting, persistent BSP inactivation. In addition, targeted insertion of the respective conditional expression cassette into a predetermined locus warrants the integrity and persistence of MDA-MB-231 clonal properties. Respective cell lines were used to investigate the influence of BSP knockdown on cellular functions *in vitro* and on tumor growth *in vivo*. In addition, microarray data for expression profiling were yielded to identify signaling cascades, which are modulated by conditional, long lasting knockdown of BSP and contribute to its mechanism of action.

## RESULTS

### Generation of cell clones

**Figure 1:**
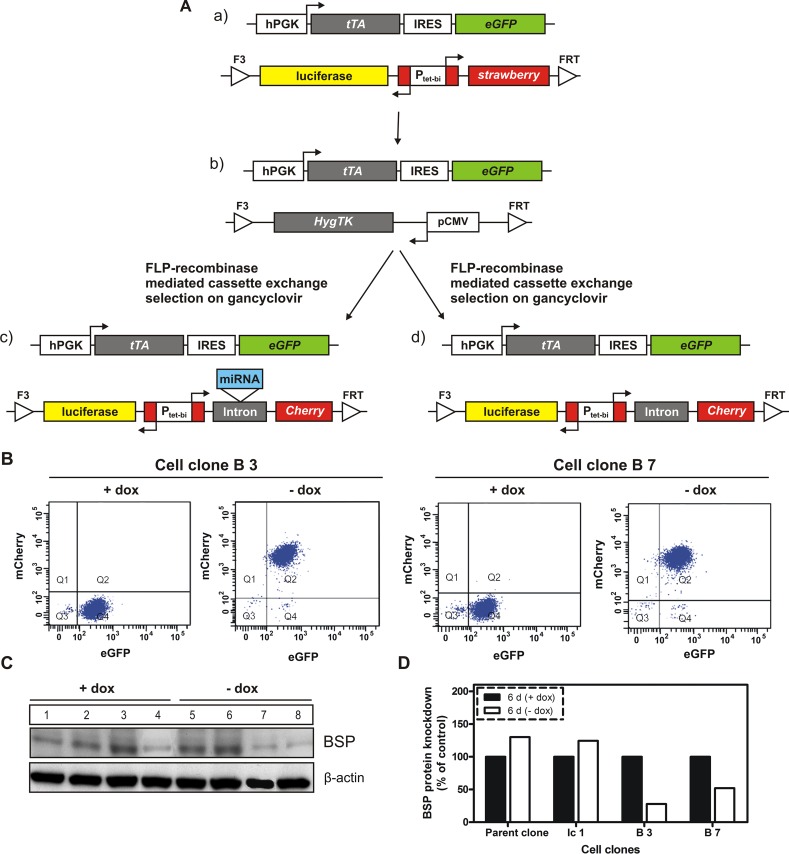
Generation of BSP knockdown and control cell clones by recombinase-mediated cassette exchange A. The parental cell clone (a) was transfected with a plasmid containing the gene cassette for hygromycin resistance and thymidine kinase (Hyg-TK), then a hygromycin resistant cell line (b) was selected for further use. Upon gancyclovir treatment, the Hyg-TK cassette of (b) was exchanged by a cassette, in which the tet-dependent bidirectional promotor P_tet-bi_ controls the simultaneous expression of reporter genes firefly luciferase and red fluorescence protein mCherry as well as the miRNA targeting BSP (c). A control cell clone without miRNA (d, termed Ic1) was generated using the same strategy. Abbreviations used: hPGK – human phosphoglycerate kinase promoter; tTA – tetracycline-controlled transactivator; IRES – internal ribosome entry site; eGFP – enhanced green fluorescent protein; luciferase – firefly luciferase; P_tet-bi_ – bidirectional tet-regulated promoter; strawberry – red fluorescent protein strawberry; HygTK – hygromycin/ thymidine kinase selection marker gene conferring resistance to hygromycin and sensitivity to ganciclovir; pCMV – cytomegalovirus promoter; FRT and F3 - wild type and mutant Flp – recombinase target sites, B. and C: Regulation of transfected genes by doxycycline. B. Flow cytometry analysis of two double transgenic cell clones; A distinct increase in mCherry expression is observed in response to cultivation of cells in media without doxycycline; Q1-Q4 indicate quadrants 1-4; eGFP - enhanced green fluorescent protein; mCherry - red fluorescent protein mCherry; C. Western blot analysis of BSP expression in control (parent and Ic 1 clones) and two specific cell clones following six days of cultivation in media with or without doxycycline; distinct inhibition of BSP production is observed in response to cultivation of cells in media without doxycycline; numbers correspond to the following experimental conditions: 1- parent cell clone in medium with doxycycline (+dox control), 2- Ic1 cell clone in medium with dox (+dox control), 3- B3 cell clone in medium with dox (+dox specific control), 4– B7 cell clone in medium with dox (+dox specific control), 5 - parent cell clone in medium without dox (-dox control), 6- Ic1 cell clone in medium without dox (-dox control), 7- B3 (miBSP) cell clone in medium without dox (-dox anti-BSP effects), 8- B7 (miBSP) cell clone in medium without dox (-dox anti-BSP effects); D. Densitometric analysis of the respective bands from Figure 1C, based on the loading control (β – actin) and normalization of the data.

To characterize the role of BSP in breast cancer, the cell line MDA – MB – 231 was used. Our parental cell clone (Figure 1Aa) constitutively expresses the tetracycline-controlled transactivator tTA (Tet-Off system) and contains an expression cassette, in which the bidirectional tet-regulated promoter P_tet-bi_ efficiently controls production of the reporter genes mStrawberry and firefly luciferase. Moreover, the tet-responsive gene expression cassette is situated in a genomic locus conferring silent but activatable tet-regulatory properties; it is flanked by Flp recombinase consensus-sequences, allowing site-directed exchange of the gene cassette, thus conserving all beneficial properties of the genomic locus and avoiding disadvantages associated with random genomic integration. Conditional BSP cell clones were generated in a two-step procedure of recombinase–mediated cassette exchange (RMCE; Figure 1A) according to Weidenfeld et al.[16]. Resulting double-transgenic MDA-MB-231 cell lines contain a tet-regulatory gene expression cassette in which P_tet-bi_ regulates mCherry and firefly luciferase gene expression. Within cell clones interfering with BSP production an artificial miRNA targeting BSP (designed according to the miRNA3 design described in Berger et al.[17]), is located within the artificial intron preceding mCherry. In absence of doxycycline, tTA drives the expression of reporter and the miRNA genes, whereas, when doxycycline is added, the drug will attach to the tTA protein, inducing a conformational change that prevents its binding to tet-regulated promoters. Genomic control and miRNA containing cell clones were generated as described in Figure 1A. The cell clones with conditional (doxycycline dependent) expression of miRNA targeting BSP were used for elucidating BSP functions and assessing whether the selected protein is an appropriate target for antimetastatic therapy.

### Characterization of cell clones

**Figure 2:**
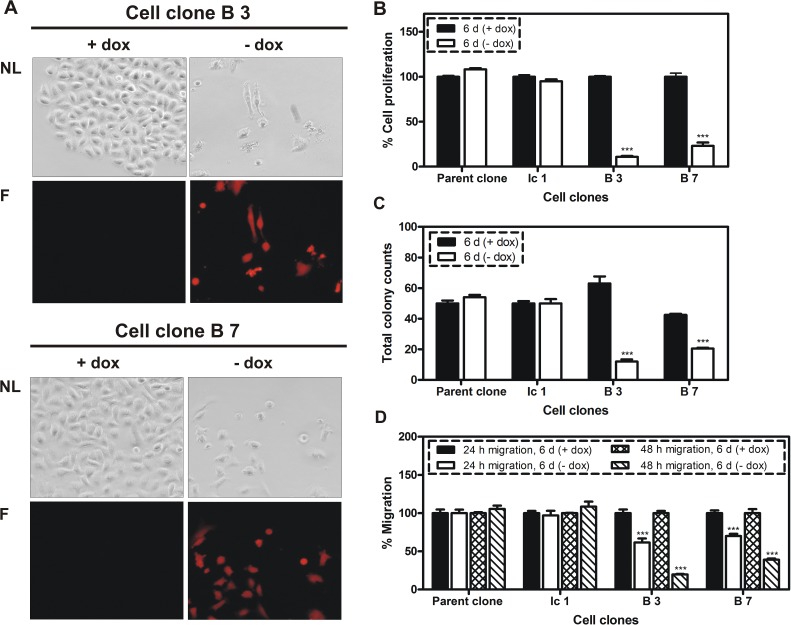
Influence of BSP knockdown on cellular properties A. Fluorescent microscopy analysis of two cell clones with conditional expression of miRNA targeting BSP in response to doxycycline (dox); triggered by the absence of dox in growth media the following changes were observed: decreased cell density, changes in cell morphology and increased expression of mCherry; NL indicates normal light, F- fluorescence; B. MTT cell proliferation assay; C. Colony formation assay; D. Migration assay. B – D: Control (parent and Ic 1 clones) and specific clones were compared following six days of cultivation in media with or without dox; A significant inhibition of proliferation, colony formation and migration was observed; *** p <0.001.

Doxycycline-dependent regulation of reporter gene mCherry was evaluated in two cell clones (designated B3 and B7) by flow cytometry analysis. As shown in Figure 1B, the cell clones display no mCherry production when tet-regulated gene expression is switched off (+dox), whereas mCherry signals increase about two orders of magnitude when tet-regulated gene expression is switched on (-dox). Next, the BSP expression was analyzed at protein level for control and miRNA containing cell clones by western blot following 6 days of cultivation in media with or without doxycycline (Figure 1 C). Here, a highly significant reduction of BSP production, ranging from 50 to 73%, was detected upon activation of tet-regulated expression (-dox) of the miRNA targeting BSP (Figure 1 D). BSP knockdown led to an observable reduction in cell number (Figure 2 A) and induced phenotypic alterations including rounding of cells and cellular fragments indicating apoptotic events. Therefore, several functional tests were performed with respective cell clones *in vitro*. miRNA containing clones showed a specific inhibition of cell proliferation (77 - 90 %; Figure 2 B), colony formation (52 - 82%; Figure 2 C) and migration (61 - 81%; Figure 2 D) upon decreased BSP production.

### Targeting extracellular BSP by anti-BSP antibody

To investigate the possibility whether a lack of secreted extracellular BSP would be responsible for the observed effect on cell proliferation, MDA-MB-231 cells were exposed to an antibody against BSP (80 to 320 μg/ml) for up to 4 days. As shown in Supplementary Figure 1A exposure to this anti-BSP antibody had no effect on their proliferation.

### Modulation of gene expression following BSP knockdown

To identify altered regulation of genes in response to BSP production inhibition, an expression profiling analysis was performed with cell clones B3 and B7, displaying 73 % and 50% reduction in BSP protein concentration upon expression of its targeting miRNA. Based on a minimum of a 2-fold modulation as cutoff for alterations in gene expression, three days of BSP knockdown in the B3 cell clone led to a change in expression of 41 genes, of which the majority (87.8%) was down-regulated. However, after 6 days of BSP knockdown, 121 genes were modulated and the majority (75.2%) was now up-regulated. In the B7 cell clone the gene modulation was examined only after 6 days of miRNA-mediated BSP inhibition and from 35 genes, 71.4% were up-regulated. The total number of genes displaying changes in expression was very low compared to all analyzed mRNAs. For categorizing altered expression levels, we differentiated between genes with significant early (day 3) modulation, which returned to control concentrations by day 6. This change in gene expression was termed “compensated short term” (Supplementary Table 1). Correspondingly, if genes showed no early modulation, but a change in expression on day 6, they were considered as “long term modulated” genes. Finally, there were genes with significant modulation observed at days 3 and 6, and this was considered as “biphasic” for those with changes from decreased to increased levels or vice versa, or as “persistent” for those with continuously increased or decreased levels. These categories were used to differentiate between short lived compensated- from persistent long term modulations under the assumption that the former will have no persistent contribution to the cells fate, as opposed to the latter. For the two time points of expression profiling, the percentage of genes displaying either long term changes (71.2%) or persistent changes (13.4%) in expression exceeded the proportion of observed biphasic changes (0.7%) or compensated short term changes (14.7%) (Supplementary Table 1). To delineate the most prominent molecular processes influenced by reduced BSP production, a 3-fold change in gene expression was used as a cutoff, which identified 27 misregulated genes in cells of the B3 cell clone and 8 in cells of the B7 clone (Table 1). There was an overlap of 5 genes, which showed 3 fold modulation in both clones, the remaining 25 were more intensively modulated in the B3 clone (n=22) or in the B7 clone (n=3). From those, the majority (18 genes) is involved in transcription processes (ontology classes transcriptional regulators, chromosomes, nuclear RNAs) implicating a major role of BSP on these intracellular events. Identified transcriptional regulators primarily act on either ER stress / apoptosis signaling cascades (DDIT3, ATF3, EGR1) or differentiation of breast epithelial cells (FOS, ID2). Minor gene ontologies modulated by BSP include the immune response, cytokines, angiogenesis, metabolism, the signal transduction and RNAs. The vast majority (27/30) of these genes was modulated more intensively in cells of the B3 clone, in line with a more severe knockdown of BSP, thus suggesting a concentration dependent effect.

**Table 1:** Functional classification of genes with more than 3fold modilation in response to miRNA- mediated BSP knockdown; comparison of the B3 and B7 cell clones

Functional classes	Gene Symbol	Gene Full Name	Fold change	
		(B 3)	(B 7)
Transcriptional regulators	FOS	FBJ murine osteosarcoma viral oncogene homolog	11.32	1.99
	DDIT3	DNA-damage-inducible transcript 3	6.52	2.43
	ATF3	activating transcription factor 3	5.01	1.93
	EGR1	early growth response 1	4.12	1.20
	CREBRF	CREB3 regulatory factor	3.31	1.82
	ID2	inhibitor of DNA binding 2, dominant negative helix-loop-helix protein	3.16	1.79
Cytokines	IL8	interleukin 8	4.93	4.14
	SCG2	secretogranin II	3.72	1.76
	IL11	interleukin 11	−3.74	−2.02
Immune response	IFIT2	interferon-induced protein with tetratricopeptide repeats 2	7.64	3.45
	KLRC2	killer cell lectin-like receptor subfamily C, member 2	4.00	2.21
Signal transduction	SERPINB2	serpin peptidase inhibitor, clade B (ovalbumin), member 2	3.61	3.81
	DDIT4	DNA-damage-inducible transcript 4	3.80	2.08
Calcium ion binding	S100P	S100 calcium binding protein P	2.58	4.27
	MEGF6	multiple EGF-like-domains 6	4.65	3.04
Angiogenesis	ANGPTL4	angiopoietin-like 4	4.39	1.92
ECM protease	MMP9	matrix metallopeptidase 9	1.01	3.30
Histones	HIST2H2AA3	histone cluster 2, H2aa3	11.26	2.66
	HIST1H2BD	histone cluster 1, H2bd	10.15	3.42
	HIST1H4A	histone cluster 1, H4a	7.59	1.10
	HIST1H1C	histone cluster 1, H1c	7.30	1.89
	HIST2H2AC	histone cluster 2, H2ac	7.13	1.99
	HIST1H2AC	histone cluster 1, H2ac	6.53	2.54
	H2AFJ	H2A histone family, member J	3.44	1.79
	HIST1H2BK	histone cluster 1, H2bk	3.05	1.01
Metabolism	CYP1B1	cytochrome P450, family 1, subfamily B, polypeptide 1	7.25	2.76
	CTH	cystathionase (cystathionine gamma-lyase)	3.10	1.73
RNA	RN7SK	RNA, 7SK small nuclear	5.93	1.91
	RNA28S5	RNA, 28S ribosomal 5	2.44	3.13
	SNORD3A	small nucleolar RNA, C/D box 3A	4.70	1.41

**Figure 3:**
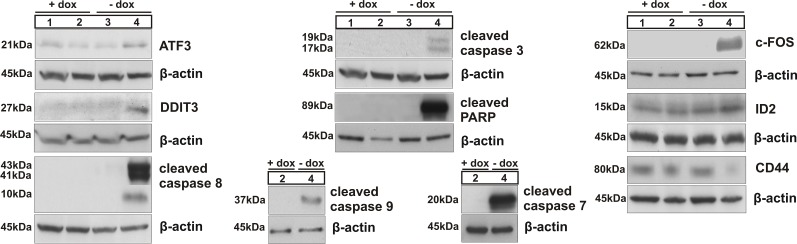
Immunoblotting of ATF3, DDIT3 (CHOP), caspases 8, 3, 9 and 7 and of PARP, related to activation of intrinsic and/or extrinsic apoptotic pathways; western blot analysis of c-FOS, ID2 and CD44; numbers correspond to the following experimental conditions: 1- parent cell clone in medium with doxycycline (+dox control), 2- B3 cell clone in medium with dox (+dox specific control), 3 - parent cell clone in medium without dox (-dox control), 4 - miBSP cell clone in medium without dox (-dox anti-BSP effects).

Western blot confirmed the changes observed for a selection of these genes (DDIT3 (CHOP), ATF3, FOS, ID2 and CD44). In addition, we observed the induction of apoptosis because of BSP inactivation, demonstrated by cleavage of caspases 8, 9, 3, 7 and of PARP (Figure 3). Collectively, these data indicate that BSP knockdown is associated with specifically altered expression of genes related to breast cancer, ER stress and apoptosis.

### Expression profiling following exposure of cell clones to an anti-BSP antibody

Both cell clones were cultivated for 6 days in media containing doxycycline and a rat monoclonal antibody against BSP (100 μg/ml) in comparison with respective cells exposed to doxycycline only. Subsequent microarray analyses of mRNA (both clones) and miRNA (B3 clone) showed that the expression of genes was identical in both clones, regardless of the antibody, when using a 2fold modulation as basis for a significant difference.

Similarly, the miRNA profiles were identical in the B3 clone cells cultivated in doxycycline containing medium with or without the antibody (Supplementary Figure 1B)

### BSP knockdown inhibits skeletal metastasis

**Figure 4:**
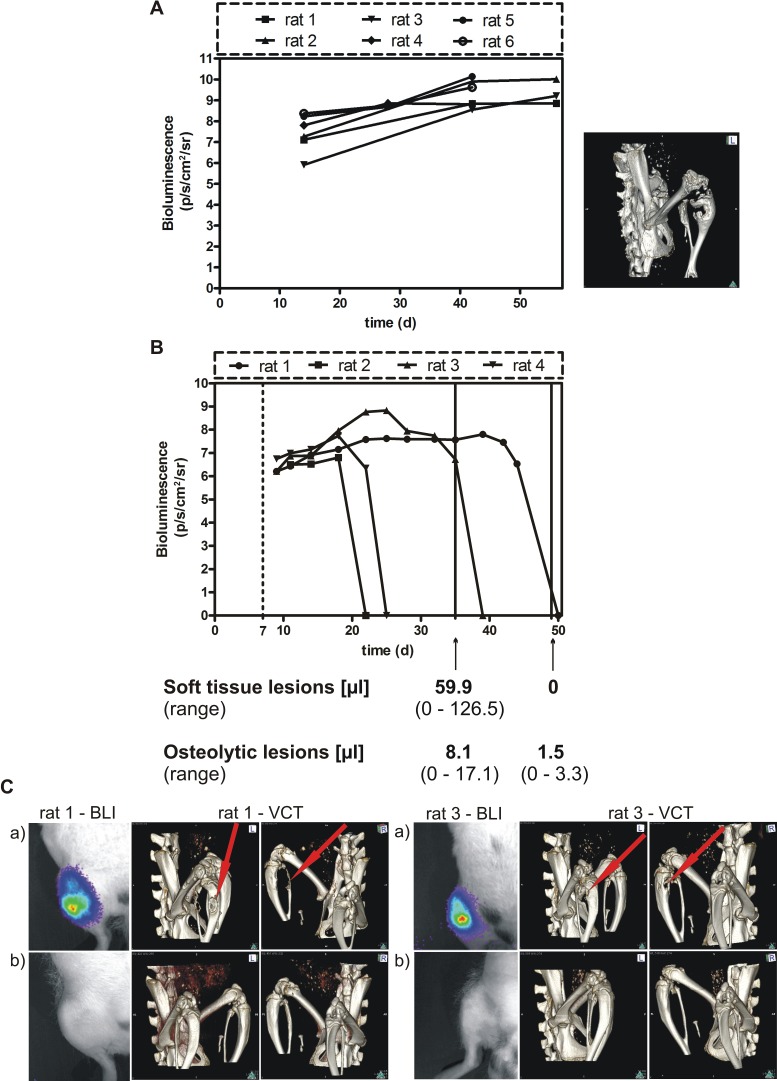
Effect of conditional BSP knockdown *in vivo* **A**. Spontaneous growth of the parental MDA-MB-231 cell line following injection into the femoral artery of nude rats. The tumor growth was estimated by BLI and the skeletal lesion monitored by VCT detection. B. BSP knockdown suppresses metastasis in vivo – bioluminescence imaging detection. B3 cells were inoculated into nude rats, which received doxycycline for 1 week (dashed line) and were exposed to miRNA treatment for up to 6 weeks; the time after tumor cell inoculation (days) is given on the x axis; bioluminescence is measured in photons/second/cm^2^/steradian, the volume of soft tissue and osteolytic lesions is indicated after 4 and 6 weeks of miRNA treatment (solid lines); C. The upper BL images (a) show the status after 4 weeks, the lower images (b) – that after 6 weeks (absence of light emission indicates absence of tumor, confirmed by histopathology); VCT scans performed after 4 weeks (upper panels (a), middle and right images) and after 6 weeks (lower panels (b), middle and right images) revealed complete remission of osteolytic lesions.

Having demonstrated that miRNA mediated inhibition of BSP expression leads to a decreased proliferation, migration and colony formation in MDA – MB – 231 cells *in vitro*, we tested the effect of BSP knockdown in an *in vivo* model of osteolytic metastasis. Parental control cells showed a steady increase in light emission and presence of osteolytic lesions within 30 days after tumor cell inoculation (Figure 4 A). Animals, which survived longer than 40 days tended to show a plateau of light emission as indication of a balance between proliferation and necrosis of tumor cells. Subsequent to these observations, controls were terminated for ethical reasons when their tumor growth reached a plateau. Here, cell clones B3 and B7 were examined in three groups of rats, respectively (Table 2). In this experiment, the expression of miRNA targeting BSP for 3 weeks led to a significant reduction of the soft tissue tumors and the osteolytic lesions in B3 and B7 cell clones, respectively, compared to the control groups (p<0.03).

**Table 2:** The effect of a conditional BSP knockdown on soft tissue and osteolytic lesions as investigated in nude rats inoculated with MDA – MB – 231 cell clones B3 and B7 into the saphenous artery

Cell clone	Period of doxycycline intake (weeks)	Period of treatment by miBSP (weeks)	Volume [μl] of soft tissue lesion (range)	T/C* 100 (soft tissue lesion)a)	Volume [μl] of osteolytic lesion (range)	T/C* 100 (skeletal lesion)a)
B 3	4	0	153.7 ^b^) (6.7 - 403.4)	100	75.4 153.7 ^b^) (0.2 - 206.9)	100
	2	2	62.8 (0 – 182.2)	41	11.3 (0 - 34.0)	15
	1	3	43.2 153.7 ^b^) (0 - 121.4)	28	5.1 153.7 ^b^) (0 - 13.2)	6.7
B 7	4	0	76.5 153.7 ^b^) (12.2- 244.1)	100	26.5 153.7 ^b^) (0.3 - 101.2)	100
	2	2	70.6 (0 - 211.8)	92	22.9 (0 - 68.0)	86
	1	3	11.0 153.7 ^b^) (0 - 30.6)	14	2.4 153.7 ^b^) (0 - 4.5)	9

a) Mean tumor or skeletal lesion volume of treated (T) over untreated control (C) rats times 100;

b) After 3 weeks of miRNA exposure, the soft tissue and osteolytic lesions were decreased significantly in treated versus control groups (p < 0.03), according to a pooled analysis with the two-tailed Wilcoxon test.

In the second experiment, the B3 cell clone was inoculated in a group of four rats to access the efficacy of a longer persistence of BSP inactivation. The tumors were monitored up to 8 weeks after tumor cell inoculation by BLI, as well as after 5 and 7 weeks by MRI and VCT. In all rats, full remissions of soft tissue and osteolytic lesions were detected (Figure 4 B and C).

Collectively, these data show a high efficiency to treat bone metastasis by inactivating BSP production *in vivo*.

## DISCUSSION

Primary breast cancer patients with elevated BSP serum concentrations were found at increased risk of developing skeletal lesions [4]. Therefore, we and others hypothesized that BSP is a potential target for treating breast cancer induced osteolytic lesions. Here, we developed a cellular model to follow this hypothesis, namely MDA – MB – 231 cell lines with the ability for a conditional knockdown of BSP, realized by combining tetracycline-controlled transcription activation (“Tet-Off system”) with RNA interference. This approach allowed for the first time full temporal control over BSP production. Persistent inhibition of endogenous BSP production resulted in a significantly reduced proliferation, migration and colony formation of respective cell clones *in vitro*. In addition, we demonstrated that reduced BSP concentrations lead to the initiation of apoptosis. The distinct gene modulation observed following impeded BSP production was not paralleled by results obtained from exposing the cells to an antibody. Similarly this antibody had no influence on the proliferation of MDA-MB-231 breast cancer cells (Supplementary Figure 1A). Corresponding to this BSP knockdown effect *in vitro*, we observed complete remissions of soft tissue and osteolytic lesions *in vivo*. Thus, the first protracted conditional knockdown of its kind revealed that BSP is a vital intracellular factor, which is necessary for tumor cell survival and growth in vivo. This assumption is strengthened by the observation that genes were more intensively modulated in cells with lower BSP concentrations, thus indicating a concentration dependent effect. Of course, only a large number of tumor cell lines will be able to mimic the properties of a certain cancer type as published for colorectal cancer cell lines recently [18]. Therefore future experiments should corroborate our results in other cell lines with affinity to the skeleton.

The shift in paradigm from BSP being a secreted protein causally involved in the generation of osteolytic lesions to being an intracellular oncogenic driver of tumor growth was suggested by our technically advanced approach in the MDA-MB-231 clones used. In preceding studies decreased expression of BSP was caused by ASOs, siRNA or shRNA [14, 15, 19]. In general, the knockdown obtained in response to these types of gene therapy was short lived or less distinct than in this study. As a consequence, only certain cellular functions like migration and invasion were clearly inhibited [15, 19]. In addition, there was some reduction in the incidence or size of osteolytic lesions, but not in soft tissue tumor volume [15, 19]. The intensive decrease in BSP expression in our study was achieved by a method, which allows conditional knockdown over a prolonged period. Furthermore, the model is applicable in cultured cells and in whole organisms [17, 20]. The established cell clones with conditional miRNA expression were generated by RMCE from a parent cell clone without anti-BSP miRNA, which was used as a control. This procedure not only conserves the appropriate tet-regulatory properties of the parental cell line, but also warrants the genome integrity, since the integration locus of the artificial gene expression cassette is predetermined and not random as with other strategies to generate stable transgenic cell lines. In contrast to studies employing a constitutive reduction in BSP expression [19], our temporal control over BSP production avoids long term adaptation to BSP deficiency, which is basis for potential survival strategies of the tumor cells.

Our model facilitated also to monitor for the first time the genome wide modulation of expression in response to inhibition of BSP production. These results are first steps in elucidating the mechanism of action of BSP knockdown leading to the induction of apoptosis, the inhibition of proliferation, migration and colony formation *in vitro*, and the anti-metastatic effects *in vivo*. From our microarray data, the exact trigger has not been determined yet. However, the increased expression of the tumor suppressor gene EGR1 (Table 1) is causal for the increase in the activating transcription factor 3 (ATF3), for EGR1 binds to the promoter region of ATF3 [21]. ATF3 is known for its role in endoplasmic reticulum (ER) induced stress [22]. In this signal cascade, increased expression of ATF4 leads not only to increased expression of DDIT3 (CHOP, GADD153, see Table 1 and Figure 3), but also to increased expression of ATF3 (Table 1 and Figure 3). This factor in turn will decrease the expression of ID1, which exerts anti-apoptotic effects [23] and will cause increased expression of its paralog ID2, as confirmed by microarray and western blot (Table 1and Figure 3), which is essential for the differentiation of breast epithelial cells. The combination of increased EGR1 levels and ER stress is known to induce apoptosis [22, 24-28]. The reduced level of EGR1 before knockdown of BSP corresponds to the observation that cancer cells often become resistant to apoptosis. However, in cells with decreased BSP concentrations, we observed induction of apoptosis, as shown by cleavage of caspases 8, 9, 3, 7 and of PARP (Figure 3), which are well-known indicators for programmed cell death [29].

Furthermore, we detected upregulation of genes that are downregulated in the primaries of breast cancer patients, including *c-FOS, EGR1* and *ID2* [30]. These proteins are transcription factors, the first two being potential tumor suppressor genes and the third promoting breast epithelial cell differentiation [24, 31, 32]. We detected a 11fold increase in *c-FOS* mRNA concentration and even stronger stimulation of its expression at protein level. However, the increase in c-FOS expression is not supposed to lead to increased formation of AP-1 transcription factor, since transcription of *c-JUN* is elevated only 1.4fold upon BSP inactivation. Therefore, c-FOS may act as a regulator of cell growth that is dependent on cell types, stimulation signals, differentiation stages, and cell cycle states [33]. In addition, published data indicate that an elevated level of c-FOS expression could precede the initiation of apoptosis in various tissues, including the prostate [31, 34].

EGR1 inhibits angiogenesis [35], suppresses breast cancer and other tumor cells' growth *in vitro* and *in vivo* [36, 37] and was significantly reduced in some cancers including breast cancer [36-41]. A decrease in EGR1 expression may play an important role in the development of breast cancer and serve as biomarker [24].

Additionally, we detected increased ID2 (inhibitor of DNA binding 2, dominant negative helix-loop-helix protein) mRNA and protein concentrations. ID2 promotes differentiation of breast epithelial cells and its reduced expression in breast cancer is associated with an unfavorable prognosis [32].

Furthermore, we found that *IL11* and *CD 44*, genes that are strongly related to metastasis formation, were decreased in response to BSP knockdown. Interleukin 11 is an osteoclast-mobilizing factor, which enables breast cancer cells to establish osteolytic metastasis in bone [42]. CD 44 is a cell-surface glycoprotein, which participates in a variety of cellular functions including tumor metastasis and is involved in many cancers, including breast cancer [43].

Finally, the urokinase-type plasminogen activator inhibitor SERPINB2 was elevated in response to BSP knockdown, which is known to be a factor of good prognosis in breast cancer patients [44].

In conclusion, the MDA – MB – 231 cell clones generated by RMCE have allowed us to investigate the effect of a conditional, prolonged knockdown of intracellular BSP. Besides the clear anti-proliferative, anti-migratory and anti-clonogenic effects observed in vitro, a curative effect was seen in rats with osteolytic lesions. These effects are probably related to the induction of apoptosis caused by modulation of ATF3 and DDIT3 (CHOP) expression in response to lowered intracellular BSP levels. This assumption needs to be confirmed in future experiments. In addition, transcription factors known as tumor suppressors or genes related to breast cancer including c-FOS, EGR1, ID2 and SERPINB2, as well as suppression of metastasis associated genes (*CD44* and *IL11*) have emerged as molecular targets of BSP knockdown. These results show that a decrease in intracellular BSP levels is likely a key mechanism for suppression of breast cancer induced skeletal lesions.

## MATERIALS AND METHODS

### Generation of plasmid constructs

The generation of the plasmids used (pF3–luc–pBI5–Intron (miRNA)–Cherry–F and pF3–luc–pBI5–Intron-Cherry–F) was a four-step process (Supplementary Figure 2). The miRNA against BSP was based on the siRNA as described before [15] (Supplementary Figure 3).

### Cell culture

Parental MDA – MB – 231 cells and subclones were cultured in RPMI 1640 medium (Invitrogen, Germany) supplemented with 10% fetal calf serum (FCS), 100ng/ml doxycycline (added to suppress tet-regulated gene expression), 2mM L-glutamine, 100 U/ml penicillin and 100μg/ ml streptomycin (Invitrogen, Germany). The cell lines were maintained in standard cell culture flasks (TPP, Switzerland) in a humidified incubator at 37°C and 5% CO_2_. They were authenticated using Multiplex Cell Authentification by Multiplexion (Heidelberg, Germany) as described recently [45]. The single nucleotide polymorphism (SNP) profiles matched known profiles.

### Recombinase mediated cassette exchange (RMCE) and generation of stably transfected cell lines

The RMCE targetable MDA-MB 231 cell line, stably transfected with hPGK- tTA- IRES-eGFP and pCMV-Hyg.TK gene cassettes (Figure 1), was cultured for 5 days in RPMI 1640 with 700 μg/ ml hygromycin B and 100 ng/ml doxycycline. Then, the hygromycin-resistant cells were further maintained for 3 days in doxycycline-containing medium, seeded into six-well plates at a density of 8 × 10^4^ and cultured for 48h before transfection.

Subsequently, these cells were transfected with 2μg of targeting plasmid pF3-luc-Ptet-Intron(miRNA)-Cherry-FRT or pF3-luc-Ptet-Intron-Cherry-FRT, 1μg of plasmid pPur (Clontech, USA), conveying puromycin resistance, and 2μg of an expression vector for Flp recombinase. Utilized vectors expressed either Flpo (mouse codon optimized recombinase) or hFlpe (“humanized” thermostable Flp gene with codon usage optimized for mammals) [46].

For plasmid transfection, X-tremeGENE 9 DNA Transfection Reagent (Roche, Germany) or Lipofectamine 2000 (Invitrogen, Germany) were used according to the manufacturers' recommendations. Transfected cells were maintained in RPMI 1640 medium for 18h, also containing puromycin (1μg/ml) and doxycycline (100ng/ml) to keep tet-regulated expression turned off. Subsequently, cells were detached by trypsin/ EDTA and transferred into 10cm dishes with standard dox-containing medium. After 24h, fresh medium was added containing ganciclovir (65 μM) for negative selection, which was maintained for 10 days. Following 4 and 6 days of cultivation in medium without doxycycline, the transfection efficiency of different pools was checked by FACS analysis. As shown in Supplementary Figure 4, the highest percentage (48.6%) of successfully transfected cells was obtained after using Xtreme GENE 9 transfection reagent and the vector expressing Flpo recombinase. Cells showing highest transfection efficiency were used for single cell deposits in 96- well plates (Sarstedt, Germany). When individual clones had reached sufficient cell numbers, they were characterized for optimal regulation of marker gene activity in response to doxycycline.

### Fluorescence microscopy and flow cytometry analysis

Selected cell clones were individually maintained for 6 days in two small culture flasks (25cm^2^) in absence or presence of doxycycline. Pictures were taken using a fluorescence microscope (Nikon ECLIPSE TE 200, Japan) with a Nikon digital sight DS-Fi1 camera. For flow cytometry analysis, the cells were harvested by trypsin/ EDTA, resuspended by PBS (Dulbeco′s Phosphate-Buffered Saline) and transferred into 5ml tubes with cell-strainer cap (Becton Dickinson Labware, USA). The FACS analyses were performed on a LSR II of the Flow Cytometry Core facility (DKFZ). They were used to ensure purity (> 98.5%) of successfully transfected cell clones and as bench mark for later comparisons.

### Immunoblotting

Immunoblotting was done essentially as described in [47] with the following modifications: a protease inhibitor cocktail (Roche, 1:25) was used and no additional dithiothreitol was added to the sample mix including loading dye. Aliquots of cell lysates (15μg) were separated by polyacrylamide gel electrophoresis (SERVA Gel neutral pH 7,4 Gradient, Serva, Germany) and electrotransferred onto polyvinylidene difluoride (PVDF) or nitrocellulose membranes (0,2μm pore size). The following proteins were detected by specific antibodies: BSP (A4217, Immundiagnostik, Bensheim), cleaved caspase 3 (#9661), cleaved caspase 8 (#9496), cleaved caspase 9 (#9501), cleaved caspase 7 (#9491), DDIT3 (#2895), cleaved PARP (#9541), c-FOS (#2250), CD44 (#3570), ID2 (#3431)(Cell signaling technologies, USA), ATF3 (sc188) and β-actin (sc1615, Santa Cruz, Heidelberg, Germany), which served as internal loading control. Horseradish peroxidase–conjugated secondary antibody included goat anti-rat (sc2006), donkey anti – goat (sc2020), goat anti - mouse (sc2055) and goat anti - rabbit (sc2054) (all from Santa Cruz, Heidelberg, Germany). Densitometric analysis was performed using the Quantity One Program (Biorad Laboratories GmbH, Munich, Germany).

### Cell proliferation, migration and colony formation assays

Proliferation of tumor cells was quantified by MTT assay. Here, 2 − 3 × 10^3^ cells/ well were seeded into 6-well plates and cultivated for 6 days in media with or without dox. The assay was performed as described [48]. For determining the influence of extracellular BSP on proliferation, MDA-MB-231 cells were exposed to a rat monoclonal antibody against BSP (Immundiagnostik, Bensheim) for 4 days, followed by MTT assay. For migration and colony formation assays, control and specific cell clones with the miRNA targeting BSP were seeded for 6 days in culture flasks with or without doxycycline containing media. For migration assays, the bottom layer of each well in 24 - well plates consisted of 700 μl RPMI 1640 medium with 10 % FCS. Then, the cells were harvested by trypsin/ EDTA and re-suspended in media without FCS and seeded (1 × 10^5^) into hanging cell culture inserts with 8 μm pore size membranes (Millicell, Millipore, Switzerland), which were transferred onto the prepared wells. After 24h, the inserts with non-migrated cells were transferred onto new wells, containing fresh medium with 10 % FCS. The migrated cells were quantified using Cell Titer Blue Reagent (Promega, Mannheim, Germany), according to the manufacturer's protocol. Following an incubation period of 4h at 37°C, the fluorescence was measured by a fluorescence reader (Synergy 2, Biotek, Germany) with excitation (560/15) and emission (590/20) filters. The tumor cell migration was followed and quantified for 2 subsequent days. For colony formation experiments, semi-solid mixtures (0.8% methylcellulose, +/ − doxycycline, 40% FCS), containing cell suspensions (1.25 × 10^3^) were carefully mixed and aliquots (400μl) were dispensed into four wells of 24 well plates. Then, they were incubated under usual cell culture conditions (37°C, 5% CO_2_ in humidified air) and colonies (> 60 cells) were counted under an inverted microscope after 7 days.

### Animals

Male nude rats (RNU strain, Charles River, Germany) were obtained at an age of 4 to 6 weeks and kept under specific pathogen free (SPF) conditions in Macrolon-IV-cages of a ventilated rack (Ventirack, UNO Roestvaststaal B.V., Zevenaar, The Netherlands) providing a 50-fold exchange of filtered air per hour as well as positive air pressure inside the cages. Constant room temperature (22 ± 1°C), air humidity (50 ± 10%) and dark-light-rhythm (12h) were maintained throughout. The animals had free access to autoclaved water and standard laboratory diet. After an adaptation period of a week, experiments were started. All animal experiments were approved by the responsible governmental animal ethics committee (Regierungspräsidium Karlsruhe, Germany).

### Ethics Statement

The investigation involving animals has been conducted in accordance with the ethical standards and according to the Declaration of Helsinki, and according to national and international guidelines and has been approved by the authors' institutional review board.

### Tumor cell inoculation

Three days prior to tumor cell inoculation, the rats received doxycycline via the drinking water (2μg/ml), which was supplemented with 240mg sodium cyclamate and 24mg saccharin-sodium per liter.

Cell clones with conditional expression of miRNA targeting BSP were cultivated in media with doxycycline. Subconfluent cells were harvested using trypsin/ EDTA, washed by PBS, subsequently counted and resuspended in PBS to a concentration of 1 × 10^5^ cells per 100μl. For tumor cell implantation, rats were anaesthetized with isoflurane (1–1.5 vol.%) in air. The cells were injected into the saphenous artery according to the procedure detailed previously with slight modification [49].

### Set up of animal experiments and tumor size determination

Initially, the two clones B3 and B7 were tested in three groups of rats (n=3, respectively), receiving continuously doxycycline (2μg/ml) for one, two or four weeks in autoclaved water. The experiment was terminated 4 weeks after tumor cell inoculation. Then, to assess late therapeutic effects, the B3 cell clone was examined in up to 8 weeks in animals (n=4), which had ingested doxycycline via the drinking water for 1 week at 2μg/ml. Bioluminescence imaging (BLI), magnetic resonance imaging (MRI) and volume computed tomography (VCT) were used to follow the tumor size in the experimental animals [49].

### Microarray analysis

Microarray analysis was accomplished as described [48]. Microarray scanning was done using an iScan array scanner. As test for significance, the student's t-test was used on the bead expression values of the two groups of interest. The average expression value was calculated as mean of the measured expressions of beads together with the standard deviation of the beads. Modulations in gene expression were considered significant, if the p-value corrected by the Benjamini-Hochberg procedure, was lower than 0.01. This was observed for all modulations exceeding an at least 2fold change.

Entry name/accession number: GSE55432 in the Gene Expression Ombnibus (GEO) database.

### miRNA Profiling

miRNA profiling was performed on RNA extracted from cells of the B3 cell clone, which had been maintained for six days in media with doxycycline and an anti–BSP antibody (100μg/ ml) or in doxycycline containing media only. To that purpose, fluorescently-labeled miRNA was prepared according to the Agilent protocol “miRNA Complete Labeling and Hyb Kit”. Labeled miRNA samples were hybridized for at least 20h at 55°C on respective chips (Agilent human miRNA Microarray Release 19.0, 8x60k). Gene Expression Microarrays were scanned using the Agilent Scanner G2505C. GeneView raw data from the scanner were analyzed with the program language R, which gives one aggregated expression value for each miRNA ID. Statistical tests (t-tests) were performed over all samples of a group in linear scale. Benjamini-Hochberg correction was applied over all p-values of the differential expression analysis.

### Statistics

The results of multiple measurements from in vitro functional tests and in vivo experiments were given as mean with corresponding standard deviation. The two-way analysis of variance (ANOVA) test, Bonferroni-posthoc test and Wilcoxon test (GraphPad-Prism5) were used to examine for independent occurrence of investigated parameters (*in vitro* functional properties and growth in vivo). A p-level less than 0.05 was considered significant.

## SUPPLEMENTARY MATERIAL FIGURES AND TABLE


