# Supplementary material for: Sustained conditional knockdown reveals intracellular bone sialoprotein as essential for breast cancer skeletal metastasis
Source: Oncotarget. 2014 Jun 26;5(14):5510–22. doi: 10.18632/oncotarget.2132 (PMC4170606; doi:10.18632/oncotarget.2132)
Supplement: Supplementary file 1 [file oncotarget-05-5510-s001.pdf]

Sustained conditional knockdown reveals intracellular bone sialoprotein as essential for breast cancer skeletal metastasis

Supplementary Material

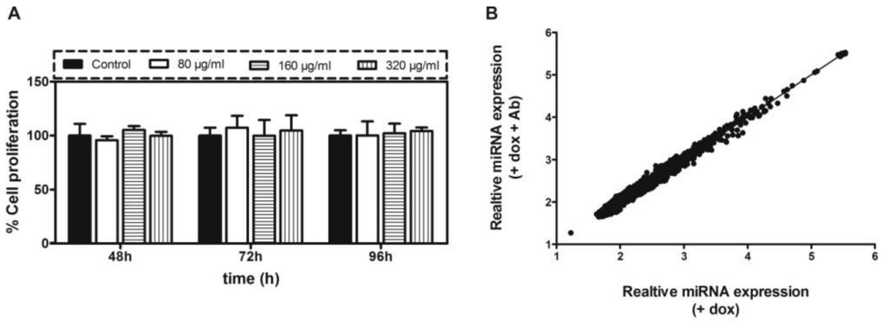

Supplementary Figure 1: Influence of a monoclonal antibody against bone sialoprotein on proliferation and miRNA expression of MDA-MB-231 breast cancer cells. A. MDA-MB-231 cells were exposed to the anti-BSP antibody for 48, 72 and 96h at concentrations of 80, 160 and 320 µg/ml, in comparison to control cells. The proliferation of cells was determined by MTT assay. B. Cells of the B3 clone (derived from MDA-MB-231 cells) were maintained in medium containing doxycycline and compared to B3 cells kept under the same conditions and exposed to the anti-BSP antibody (100 µg/ml) for six days. The RNA of both flasks was compared for their miRNA profiles by micro-array analysis (Agilent). The resulting expression levels were plotted against each other.

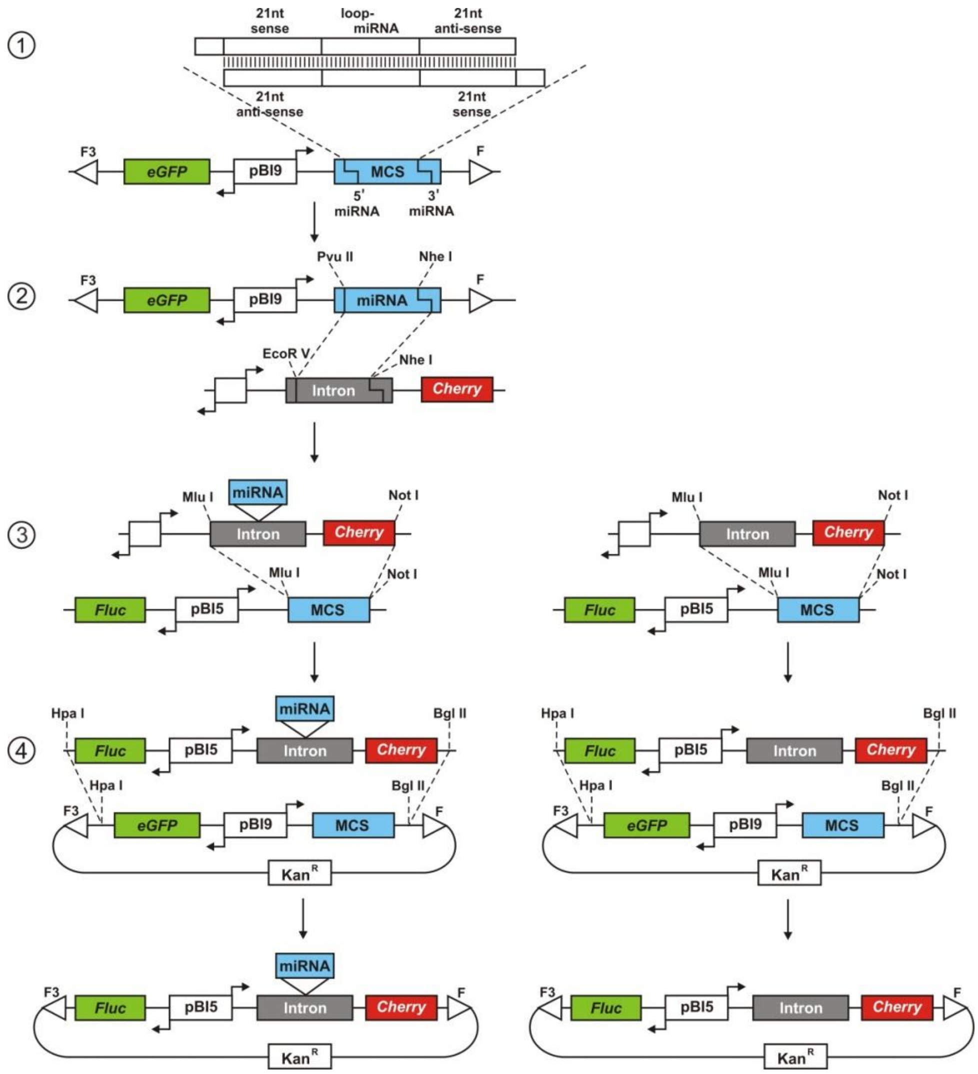

Supplementary Figure 2: Generation of the plasmids pF3–luc–pBI5–Intron (miRNA)–Cherry–F and pF3–luc–pBI5–Intron-Cherry–F in a four step process. The miRNA targeting BSP was generated by annealing two complementary oligonucleotides. In the first step, the annealed oligonucleotides were integrated into the multiple cloning site (MCS) of pBI9e.F3.eGFP.MCS by using two BsaI restriction sites. Next, the miRNA was released from the plasmid pBI-9.F3.eGFP.miRNA as a fragment with blunt PvuII and sticky NheI sites and ligated with a vector fragment from pIntron.Cherry with a blunt EcoRV and a sticky NheI site, yielding pIntron(miRNA).Cherry. In the third step, the reading frames of Intron(miRNA)-Cherry or Intron-Cherry were excised from this plasmid or pIntron.Cherry using the enzymes MluI and NotI and were cloned into the MCS of Fluc-pBI5-MCS. In the last step, the gene cassettes Fluc-pBI5-Intron(miRNA)-Cherry or Fluc-pBI5-Intron-Cherry were released from the respective plasmids using HpaI and BglII restriction sites and the resulting fragments were integrated into pBI9e.F3.eGFP.MCS, thus yielding F3-Fluc-pBI5-Intron(miRNA)-Cherry-F or F3-Fluc-pBI5-Intron-Cherry-F. Abbreviations used: FRT and F3 - wild type and mutant Flp – recombinase target sites,eGFP - enhanced green fluorescent protein; Fluc – firefly luciferase; Cherry – red fluorescence protein mCherry, Kan – gene for kanamycin resistance.

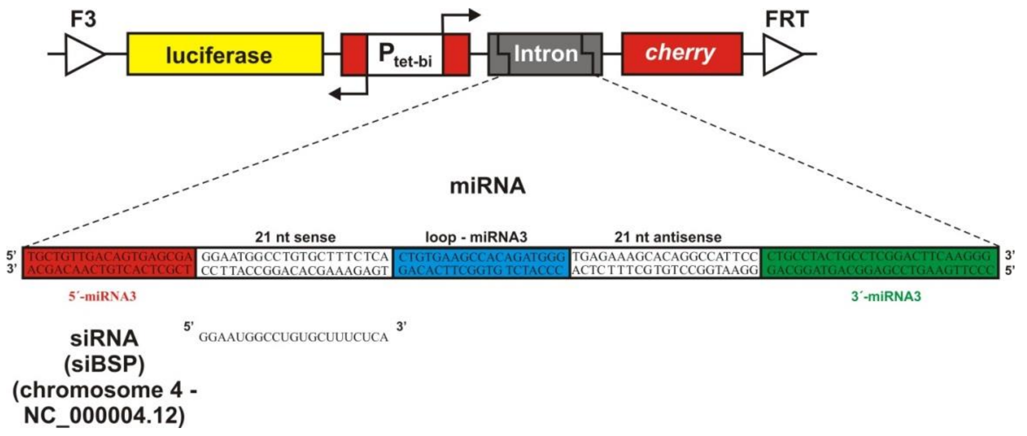

Supplementary Figure 3: Details of the gene cassette, containing an artificial miRNA directed against BSP. The letter code shows the miRNA and siRNA sequences, which target BSP. The artificial miRNA was integrated into an intron sequence in the Ptet - gene cassette. Abbreviations used: FRT and F3 - wild type and mutant Flp – recombinase target sites, luciferase - firefly luciferase; $P_{tet\text{-}bi}$ – bidirectional tet-regulated promoter, cherry – red fluorescence protein mCherry.

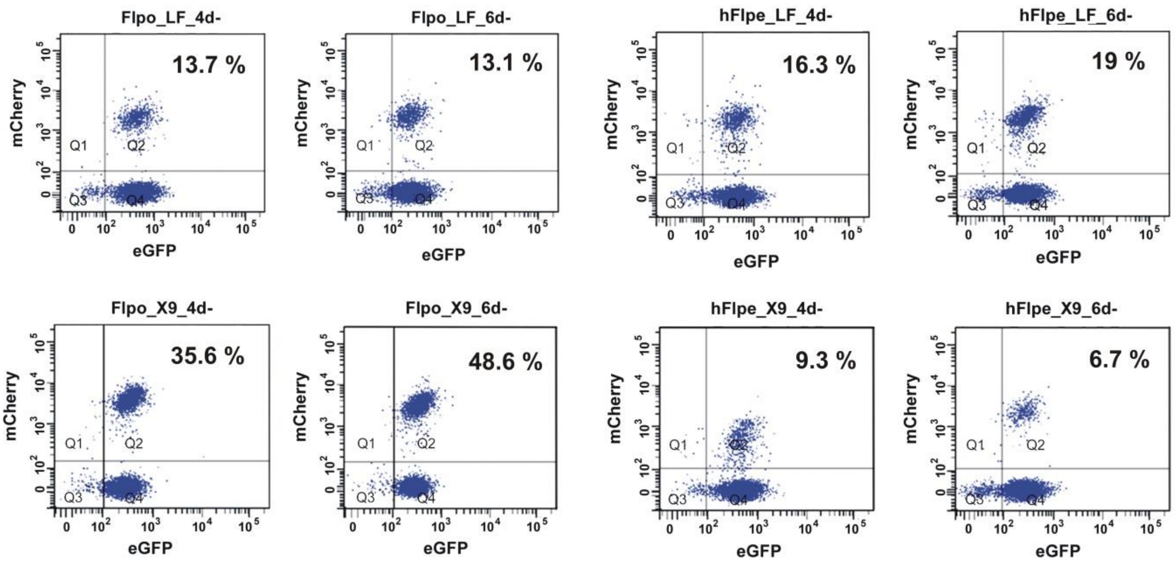

Supplementary Figure 4: Efficacy of RMCE assessed by flow cytometry. The percentages of cells positive for GFP and mCherry are given. Abbreviations used: LF - lipofectamine 2000, X9 - Xtreme GENE 9; Flp – flippase; Flpo - mouse codon optimized recombinase, hFlpe - "humanized" thermostable Flp with codon usage optimized for mammals; 4d-, 6d- four or six days of cultivation in medium without doxycycline.

Supplementary Table 1: Description of gene expression modulation observed in cells of B3 cell clone.

Changes in gene expression	Significant modulation [a)] day 3	day 6	Number of genes	Percentage of genes with more than 2fold change in expression
Compensated short term	↓ [b)]	- [d)]	18	12.6
	↑ [c)]	-	3	2.1
Biphasic	↓	↑	1	0.7
	↑	↓	0	0
Persistent	↓	↓	17	12
	↑	↑	2	1.4
Long term	-	↓	13	9.2
	-	↑	88	62

[a)] Fold change in expression > 2.0 and < - 2.0
[b)] Significant downregulation
[c)] Significant upregulation
[d)] A less than 2fold modulation of gene expression was disregarded
